# Supplementary material for: Brain dopamine responses to ultra-processed milkshakes are highly variable and not significantly related to adiposity in humans
Source: medRxiv. 2024 Jun 25:2024.06.24.24309440. Preprint. [Version 1] doi: 10.1101/2024.06.24.24309440 (PMC11302720; doi:10.1101/2024.06.24.24309440)
Supplement: 1 [file NIHPP2024.06.24.24309440V1-supplement-1.pdf]

medRxiv preprint doi: https://doi.org/10.1101/2024.06.24.24309440; this version posted June 25, 2024. The copyright holder for this preprint (which was not certified by peer review) is the author/funder, who has granted medRxiv a license to display the preprint in perpetuity.
This article is a US Government work. It is not subject to copyright under 17 USC 105 and is also made available for use under a CC0 license.

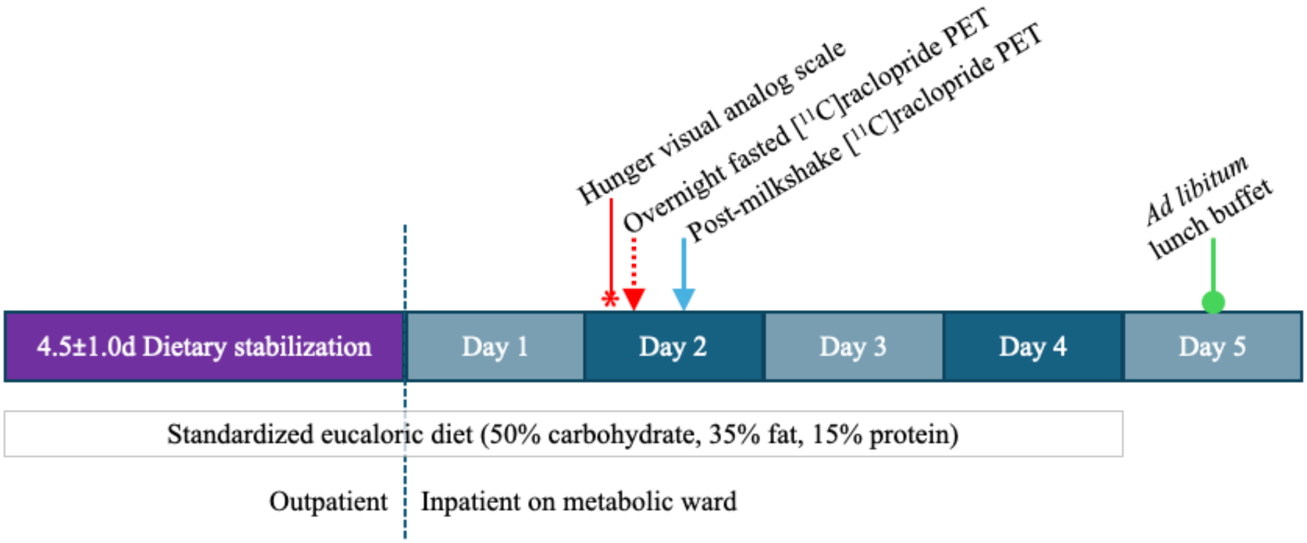

Supplementary Figure 1. Study design. Participants (n=50) consumed the provided weight-stabilizing standardized diet for an average of 4.5±1.0 days (mode 5 full days) prior to admission to the NIH Clinical Center for testing. During their inpatient stay, participants continued their dietary stabilization. [^{11}C]Raclopride displacement scan protocol was conducted on pseudo randomly assigned day during inpatient stay (2.4±0.9 days; mode 2 days), after approximately 6.8±1.1 total days (mode 7 full days) of dietary stabilization. Participants completed a confirmed overnight fast (~15 h) at which time hunger was assessed via digital visual analog scale prior to their first [^{11}C]raclopride scan. Upon completion, participants rested quietly in an adjacent room for roughly 75 minutes, at which time they consumed 226mL vanilla milkshake within 5 minutes and began their second and final [^{11}C]raclopride scan approximately 30 minutes after consuming the milkshake. On the final day of their inpatient stay, participants were presented with an ad libitum lunch buffet after a confirmed overnight fast.

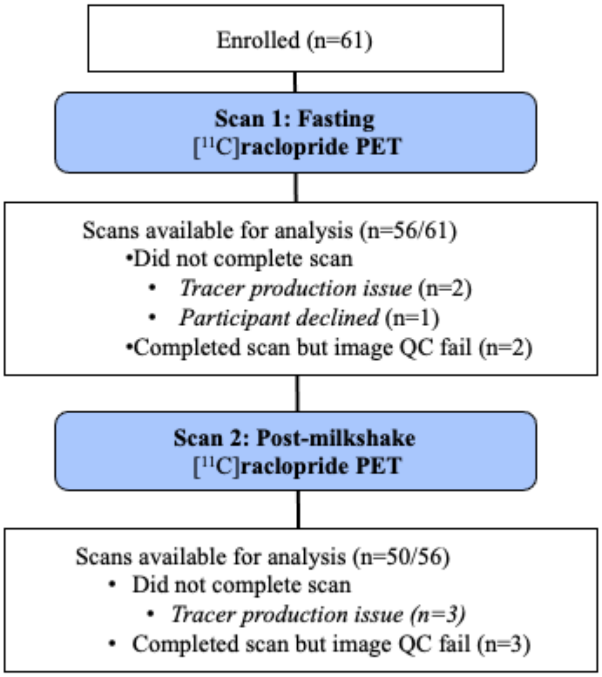

Supplementary Figure 2. Enrollment and data distillation details. Sixty-one participants provided informed consent for enrollment in this preregistered clinical trial. Only the sample numbers pertinent to the current analysis for primary outcomes are presented here.

97

medRxiv preprint doi: https://doi.org/10.1101/2024.06.24.24309440; this version posted June 25, 2024. The copyright holder for this preprint (which was not certified by peer review) is the author/funder, who has granted medRxiv a license to display the preprint in perpetuity.
This article is a US Government work. It is not subject to copyright under 17 USC 105 and is also made available for use under a CC0 license.

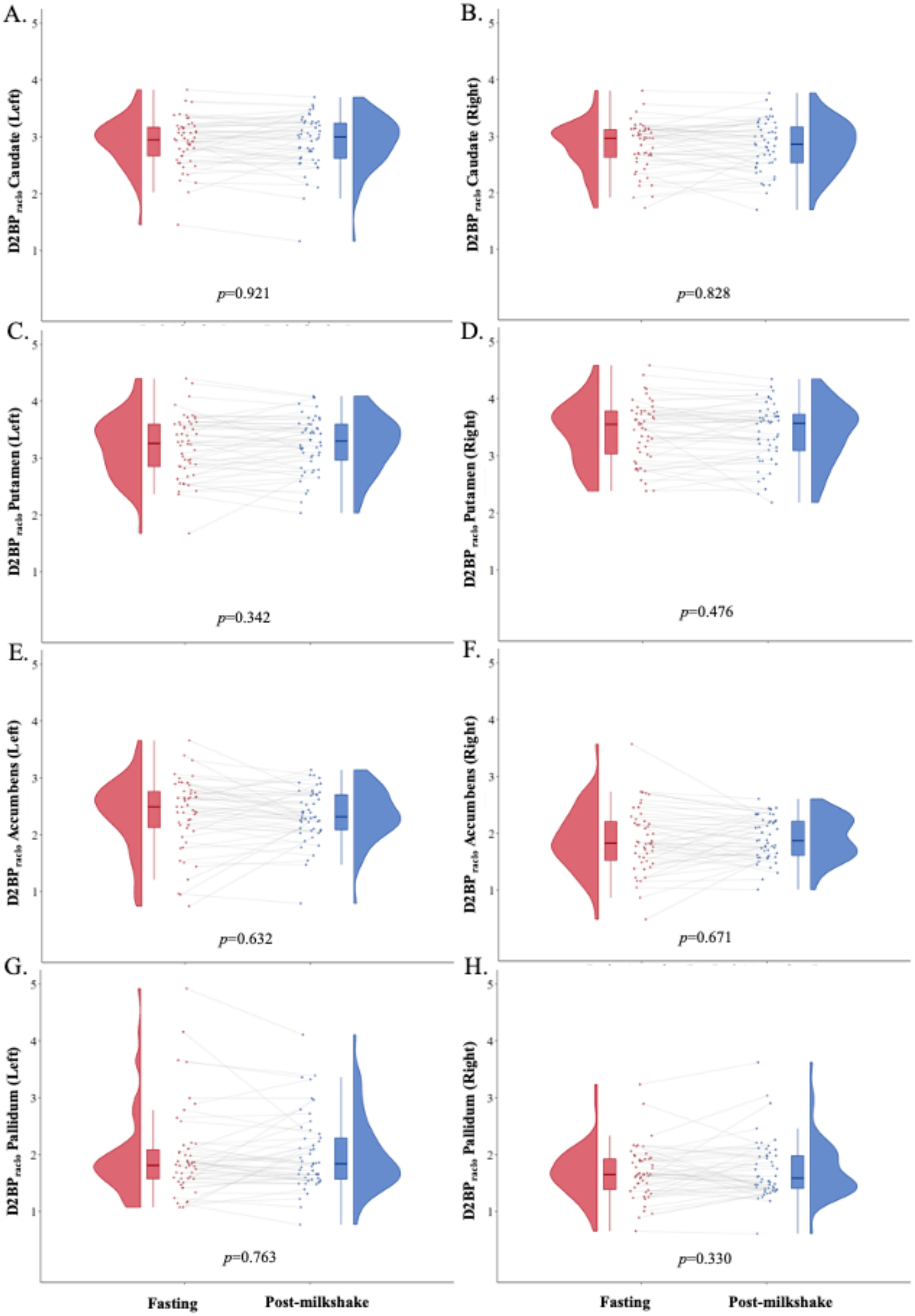

Supplementary Figure 3. An ultra-processed milkshake did not significantly impact [^{11}C]raclopride binding potential across the whole sample (n=50) in striatal sub regions of interest: (A) left caudate, (B) right caudate, (C) left putamen, (D) right putamen, (E) left accumbens, (F) right accumbens, (G) left pallidum, and (H) right pallidum.

medRxiv preprint doi: https://doi.org/10.1101/2024.06.24.24309440; this version posted June 25, 2024. The copyright holder for this preprint (which was not certified by peer review) is the author/funder, who has granted medRxiv a license to display the preprint in perpetuity.
This article is a US Government work. It is not subject to copyright under 17 USC 105 and is also made available for use under a CC0 license.

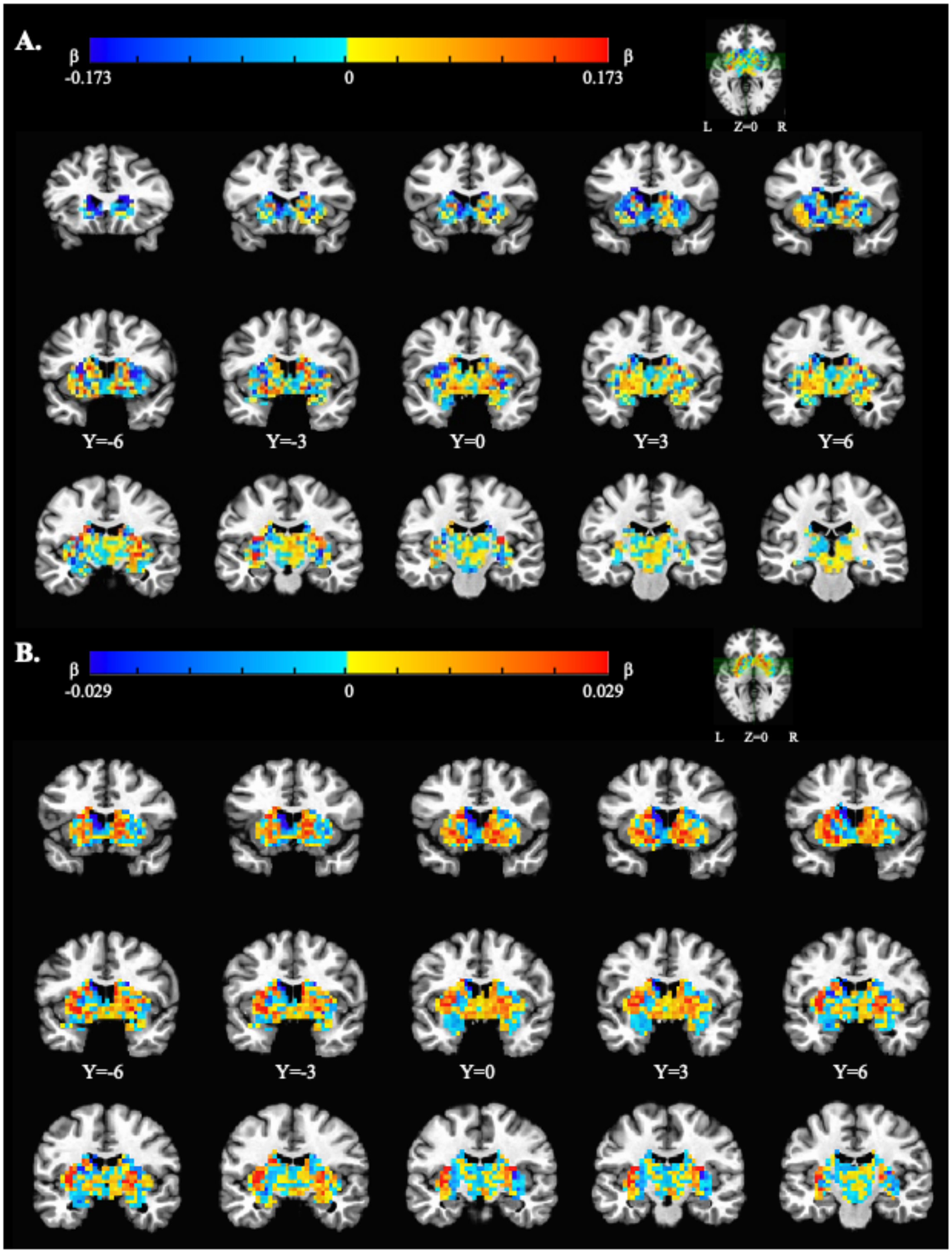

Supplementary Figure 4. (A) Response to milkshake across 50 adults. Unthresholded beta maps contrasting D2BP post-milkshake vs D2BP fasting, using striatal mask. AFNI 3dANOVA2. No clusters survive *a priori* correction for multiple comparisons (NN=1, k_e=20, p_{uncorr}=0.1) **(B) Correlation between BMI and milkshake response (Δ** D2BP fasting – post-milkshake) across 50 adults. Unthresholded beta maps. AFNI 3dttest++. No clusters survive *a priori* correction for multiple comparisons (NN=1, k_e=20, p_{uncorr}=0.1).

medRxiv preprint doi: https://doi.org/10.1101/2024.06.24.24309440; this version posted June 25, 2024. The copyright holder for this preprint (which was not certified by peer review) is the author/funder, who has granted medRxiv a license to display the preprint in perpetuity.
This article is a US Government work. It is not subject to copyright under 17 USC 105 and is also made available for use under a CC0 license.

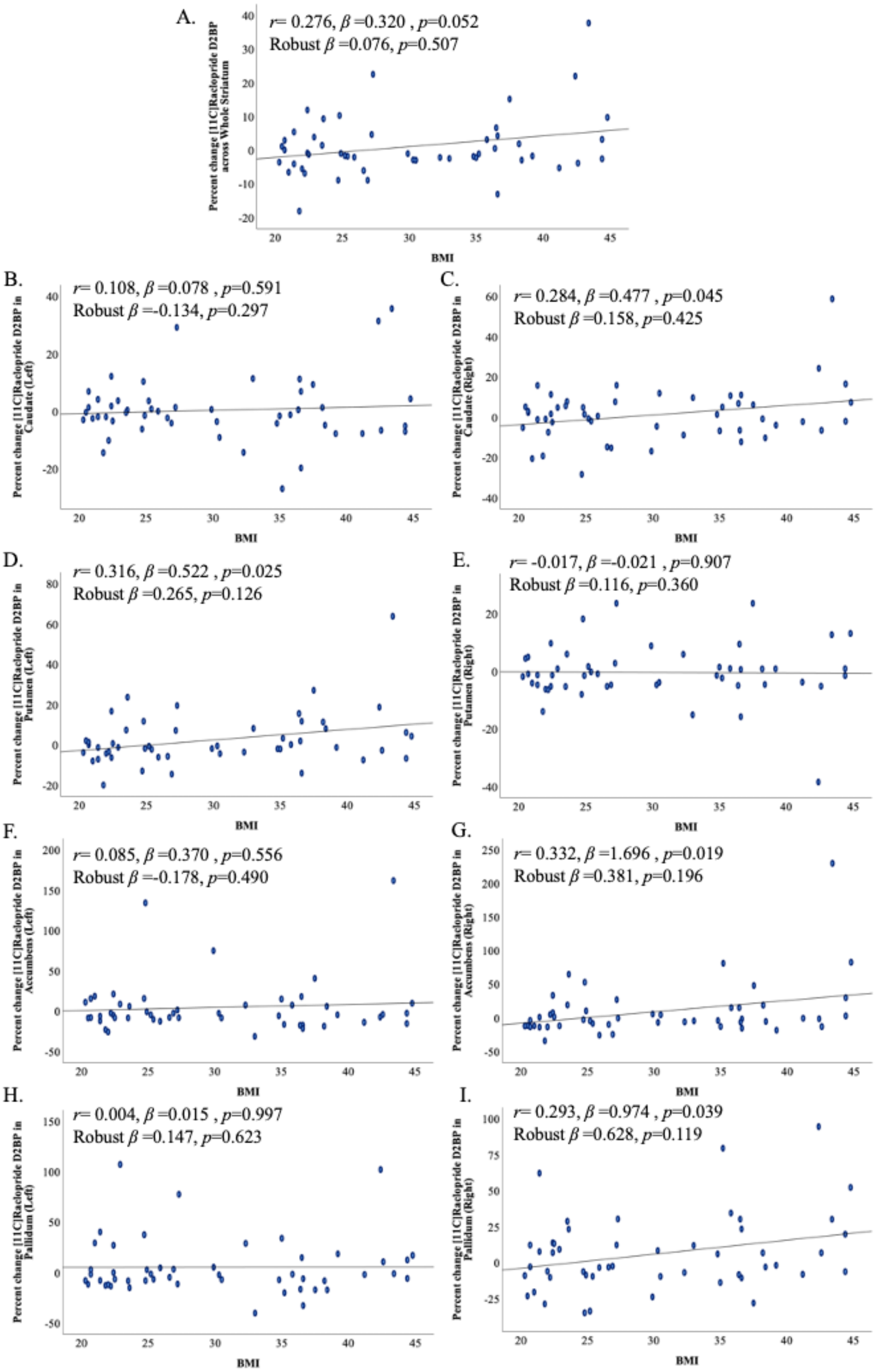

Supplementary Figure 5. Relationships between BMI and response to milkshake (% change D2BP from fasting) across (A) whole striatum and striatal subregions (B – I) are not robust to influential data points.

medRxiv preprint doi: https://doi.org/10.1101/2024.06.24.24309440; this version posted June 25, 2024. The copyright holder for this preprint (which was not certified by peer review) is the author/funder, who has granted medRxiv a license to display the preprint in perpetuity. This article is a US Government work. It is not subject to copyright under 17 USC 105 and is also made available for use under a CC0 license.

Supplementary Table 1. Locations of striatal clusters with significant correlations. PET resolution 3.5mm³. Imaging analyses conducted in Analysis of Functional Neuroimaging (AFNI) within striatal region binding potential mask. Clusters defined by voxels with faces touching, cluster extent of 20, bi-sided p_{uncorr}<0.1.

	Location of peak			Voxels	Size (mm³)	t-stat	alpha
	x	y	z				
Δ D2BP (Post milkshake – Fasting) [(a)]							
No clusters	--	--	--	--	--	--	--
Δ D2BP x BMI [(b)]							
No clusters	--	--	--	--	--	--	--
Δ D2BP x Fasting Hunger [(c)]							
Left putamen	22.8	-6.0	13.5	106	4545	-2.44	<0.01
Right caudate	-15.8	-20.0	6.5	39	1672	-2.46	<0.05
Right putamen	-33.2	11.5	-0.5	25	1072	2.53	>0.10
Right pallidum	-15.8	-2.5	-0.5	20	858	-2.69	>0.10
Δ D2BP x Ad Libitum Total Energy Intake[(d)]							
Left putamen	26.2	-2.5	3.0	33	1415	-3.74	>0.05
Δ D2BP x Ad Libitum Non-cookie Energy Intake[(d)]							
No clusters	--	--	--	--	--	--	--
Δ D2BP x Ad Libitum Cookie Energy Intake[(d)]							
Left putamen	29.8	11.5	6.6	41	1757	-2.85	<0.02
Right putamen	-26.2	11.5	6.5	34	1458	-2.52	0.05

a. Paired samples t-test, n=50
b. 1 sample t-test, n=50
c. 1 sample t-test, n=45
d. 1 sample t-test, n=45

medRxiv preprint doi: https://doi.org/10.1101/2024.06.24.24309440; this version posted June 25, 2024. The copyright holder for this preprint (which was not certified by peer review) is the author/funder, who has granted medRxiv a license to display the preprint in perpetuity. This article is a US Government work. It is not subject to copyright under 17 USC 105 and is also made available for use under a CC0 license.

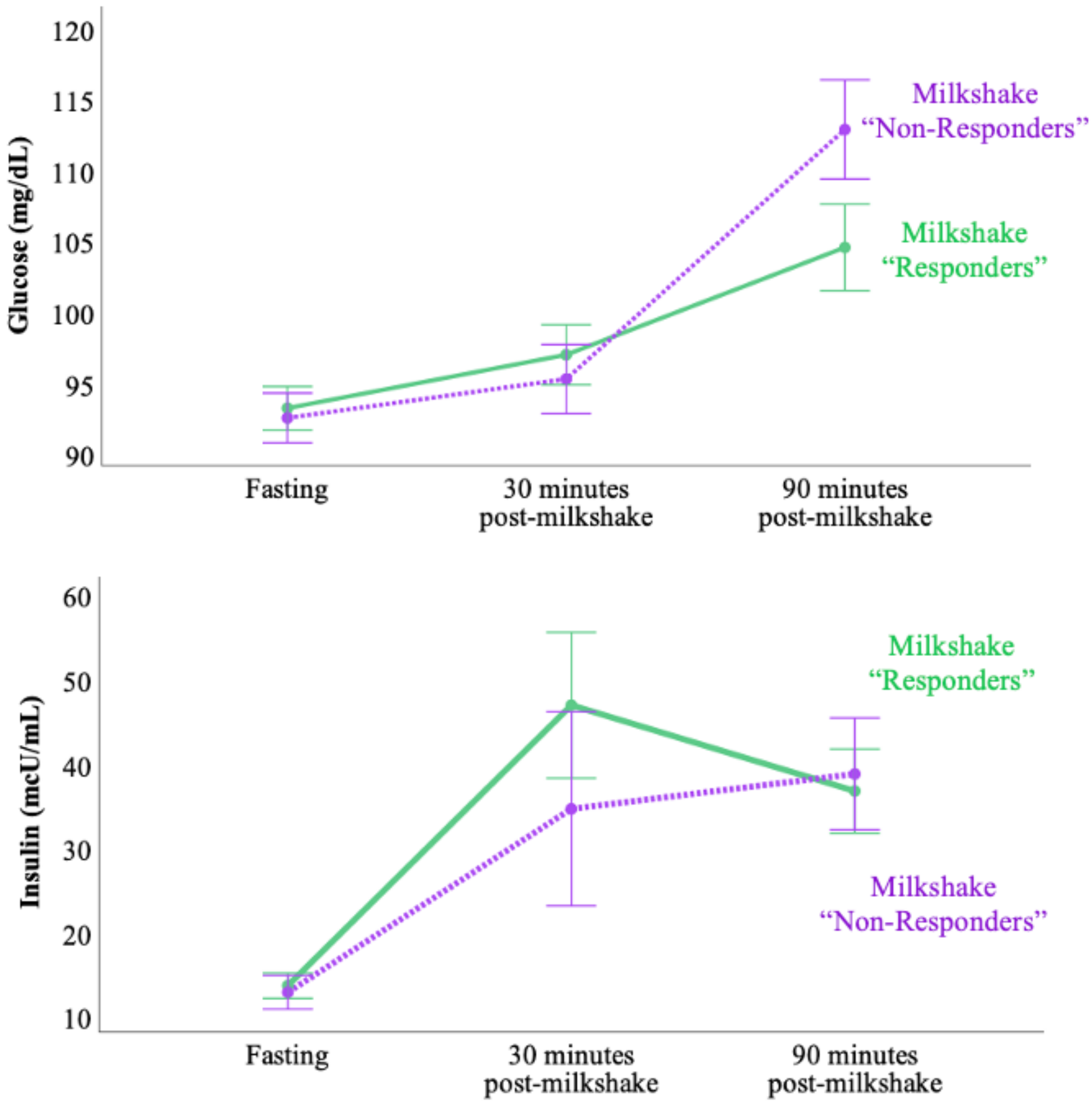

Supplementary Figure 5. **Glycemic and insulinemic response to milkshake.** Overall, milkshake caused a significant increase from fasting levels of both glucose (F=27.0, p<0.001, n=44) and insulin (F=25.4, p<0.001, n=36) over the duration of the scan. However, the interaction between time and dopamine response (group) was not significant for either glucose (F=2.2, p=0.125, n=44) or insulin responses (F=0.75, p=0.480, n=36). Error bars represent standard error.

medRxiv preprint doi: https://doi.org/10.1101/2024.06.24.24309440; this version posted June 25, 2024. The copyright holder for this preprint (which was not certified by peer review) is the author/funder, who has granted medRxiv a license to display the preprint in perpetuity.
This article is a US Government work. It is not subject to copyright under 17 USC 105 and is also made available for use under a CC0 license.

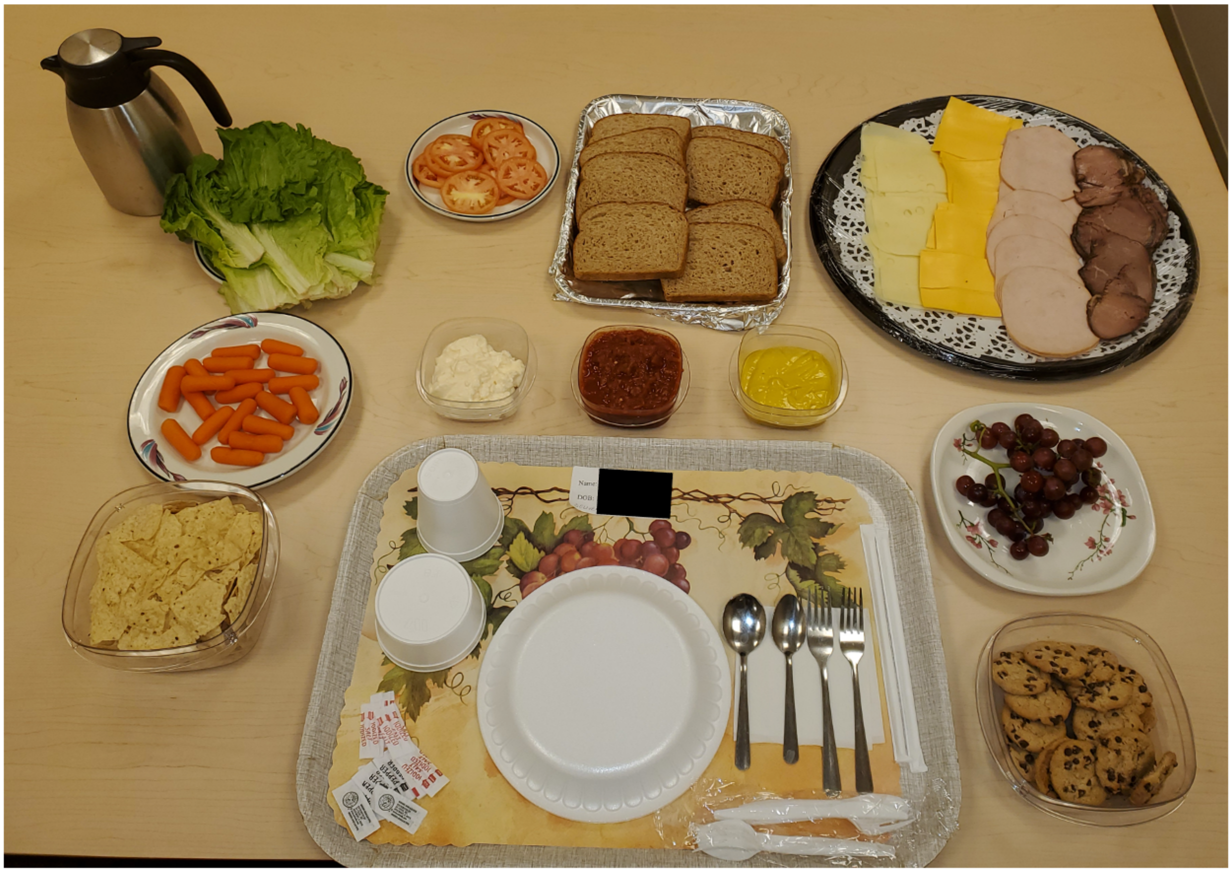

Supplementary Figure 6. Ad libitum buffet array offered for lunch (~12:00pm) after an overnight fast on the day of their discharge. Participants were presented with the above meal (>6000 kcal, 35% carbohydrate, 17% protein, 48% fat) and instructed to consume as much or as little as they wanted. Each food was weighed before and after consumption to determine total nutrient intake. Participants were presented with: 8 slices of Ultimate Grains Whole Wheat Bread, 250g roast beef deli meat, 250g turkey deli meat, 220g Glenview Farms Swiss Cheese, 220g Glenview Farms American cheese, 200g sliced tomatoes, 200g green leaf lettuce, 200g grapes, 18 Chips Ahoy! chocolate chip cookies, 135g Hellmann's Real mayonnaise, 135g Monarch yellow mustard, 375g Pasado mild salsa, 200g baby carrots, 180g Tostito tortilla chips, and 850g water. (Bread and cookies were weighed before array administration and the weight was recorded in grams.)
